# Angiotensin II upregulates the expression of placental growth factor in human vascular endothelial cells and smooth muscle cells

**DOI:** 10.1186/1471-2121-11-36

**Published:** 2010-05-26

**Authors:** Pingxi Pan, Hua Fu, Lingjun Zhang, He Huang, Fengming Luo, Wenchao Wu, Yingqiang Guo, Xiaojing Liu

**Affiliations:** 1 Laboratory of Cardiovascular Diseases, National key Laboratory of Biotherapy of Human Diseases, West China Hospital, Sichuan University, Chengdu 610041, China; 2 Department of Cardiology, West China Hospital, Sichuan University, Chengdu 610041, China; 3 Golden-card ward, West China Hospital, Sichuan University, Chengdu 610041, China; 4 Department of Thoracic & Cardiovascular surgery, West China Hospital, Sichuan University, Chengdu 610041, China

## Abstract

**Background:**

Atherosclerosis is now recognized as a chronic inflammatory disease. Angiotensin II (Ang II) is a critical factor in inflammatory responses, which promotes the pathogenesis of atherosclerosis. Placental growth factor (PlGF) is a member of the vascular endothelial growth factor (VEGF) family cytokines and is associated with inflammatory progress of atherosclerosis. However, the potential link between PlGF and Ang II has not been investigated. In the current study, whether Ang II could regulate PlGF expression, and the effect of PlGF on cell proliferation, was investigated in human vascular endothelial cells (VECs) and smooth muscle cells (VSMCs).

**Results:**

In growth-arrested human VECs and VSMCs, Ang II induced PlGF mRNA expression after 4 hour treatment, and peaked at 24 hours. 10^-6 ^mol/L Ang II increased PlGF protein production after 8 hour treatment, and peaked at 24 hours. Stimulation with Ang II also induced mRNA expression of VEGF receptor-1 and -2(VEGFR-1 and -2) in these cells. The Ang II type I receptor (AT_1_R) antagonist blocked Ang II-induced PlGF gene expression and protein production. Several intracellular signals elicited by Ang II were involved in PlGF synthesis, including activation of protein kinase C, extracellular signal-regulated kinase 1/2 (ERK1/2) and PI3-kinase. A neutralizing antibody against PlGF partially inhibited the Ang II-induced proliferation of VECs and VSMCs. However, this antibody showed little effect on the basal proliferation in these cells, whereas blocking antibody of VEGF could suppress both basal and Ang II-induced proliferation in VECs and VSMCs.

**Conclusion:**

Our results showed for the first time that Ang II could induce the gene expression and protein production of PlGF in VECs and VSMCs, which might play an important role in the pathogenesis of vascular inflammation and atherosclerosis.

## Background

Atherosclerosis is now considered to be a chronic inflammatory process which may ultimately lead to acute myocardial infarction, cerebrovascular and peripheral vascular diseases [1,2]. Plenty of data suggest that the rennin-angiotensin system (RAS) plays an important role in the development of many cardiovascular diseases, including the pathophysiological process of atherosclerosis [3,4]. Many studies have shown that inhibition of the RAS could reduce inflammation and oxidative stress [5]. Angiotensin II (Ang II), one of the major effectors of the RAS, is a cytokine that regulates cell growth, inflammation and fibrosis contributing to the progression of vascular damage [4,6,7]. Ang II participates in atherosclerosis pathogenesis by inducing inflammation and apoptosis, facilitating absorption of oxidative low density lipoprotein, generating oxygenic radicals and impacting fibrinolysis function [7]. The physiological actions of Ang II are mediated via its type 1 receptor (AT_1_R) and type 2 receptor (AT_2_R), which are expressed under different developmental, tissue-specific, and disease-specific conditions [8,9]. It has been shown that Ang II activates NF-κB, a key component of inflammation, in vascular smooth muscle cells (VSMCs)[10]. However, the exact mechanism of Ang II-mediated inflammation in vascular endothelial cells (VECs) or VSMCs is still largely unclear.

Recently, Placenta growth factor (PlGF) has emerged as a key factor in vascular inflammation and progression of atherosclerosis [11-13]. PlGF is a member of the vascular endothelial growth factor (VEGF) family cytokines and is associated with inflammation and with pathologic angiogenesis [14-16]. It is a polypeptide growth hormone that binds to Flt-1-receptor (VEGFR-1), neuropilin-1 (NRP1) and neuropilin-2 (NRP2) receptors, but not to VEGF-receptor type 2 (VEGFR-2)[14]. Recent study has shown that PlGF is required for macrophage infiltration in early atherosclerotic lesions in apolipoprotein E-deficient mice [11]. PlGF has atherogenic properties including recruitment and adhesion of monocytes, induction the production of proteinase, and thrombus formation through stimulating tissue factor secretion [12]. It is up-regulated in early and advanced atherosclerotic lesions, acting as a primary inflammatory instigator of atherosclerotic plaque instability[12]. Moreover, it has been recognized as an independent biomarker of adverse outcome in patients with acute coronary syndromes (ACS) [13,17]. As a more specific marker of vascular inflammation, PlGF might be considered for risk stratification of patients with ACS[13]. The PlGF expression can be induced by hypoxia and various pro-inflammatory stimuli [18]. This induction is mediated via NF-kappa B and metal response transcription factor-1(MTF-1)[19]. However, the regulation of PlGF expression in vascular cells, and its mechanisms of action have received little attention in atherosclerosis research.

Because PlGF plays a role in initiation and progression of atherosclerosis, it is interesting to examine the potential interaction between Ang II and PlGF. However, the connection between Ang II and PlGF expression in vascular cells has not been studied. In this study, we examined the effect of Ang II on the PlGF expression in both human VECs and VSMCs. Previously, the HUVEC-derived endothelial cell line (EA.Hy 926) [20,21] and human umbilical artery smooth muscle cells (HUASMCs)[22,23] have been characterized as models of investigating the functions of VECs and VSMC, respectively. EA.Hy 926 endothelial cells and HUASMCs were used in the present study.

## Results

### Angiotensin II increases PlGF mRNA and protein levels in both EA.Hy 926 cells and HUASMCs

The human umbilical vein endothelial cell-derived cell line EA.Hy 926 was used in this study. As an established cell line, EA.Hy 926 cell is homogenous compare to the variable primary cells from individual donors.

**Figure 1:**
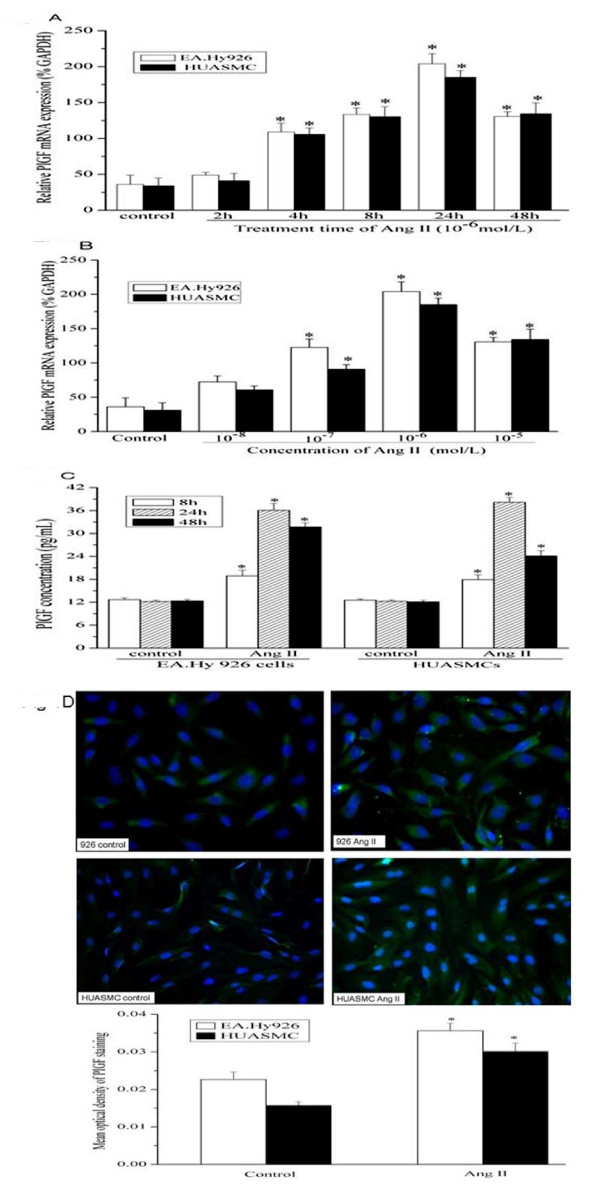
**Angiotensin II increases PlGF mRNA expression (A, B) and protein production (C, D) in EA.Hy 926 endothelial cells and HUASMCs**. Growth-arrested EA.Hy 926 cells and HUASMCs were stimulated with Angiotensin II (10^-6 ^mol/L) for different durations. **(A)** Quantitative RT-PCR (Q-PCR) results. Total RNA was isolated from cells. After reverse transcription, they were subjected to quantitative PCR analysis to determine PlGF mRNA level. Graph is representative of relative PlGF mRNA levels in the various conditions. Experiments were performed five times with the similar results (n = 5 in each group). * indicates *P *< 0.05 *vs *control EA.Hy 926 cells or HUASMCs. **(B)** Induction of PlGF mRNA expression is Ang II concentration-dependent. * indicates *P *< 0.05 *vs *control EA.Hy 926 cells or HUASMCs. **(C)** Release of PlGF protein measured by ELISA. Cells were incubated with Ang II (10^-6 ^mol/L) for 8, 24 or 48 h, and PlGF protein released into cell culture media was measured by ELISA (n = 3 in each group). * indicates *P *< 0.05 *vs *control EA.Hy 926 cells or HUASMCs. **(D)** Expression of PlGF protein detected by immunofluorescence assay. Immunofluorescence detecting of PlGF expression in untreated control cells, and cells stimulated with Ang II (10^-6 ^mol/L) for 24 hours (original magnification of 200 ×). Mean optical density (MOD) of PlGF staining was measured on the images by using the ImageJ software. The figure shows one of three similar experiments. * *P *< 0.05 *vs *control EA.Hy 926 cells or HUASMCs.

To determine whether Ang II modulates PlGF gene expression in VECs, EA.Hy 926 endothelial cells were treated with different concentrations of Ang II. Total RNA was isolated at various time points (up to 48 hours). After 4 hour treatment, real-time quantitative RT-PCR revealed that 10^-6 ^mol/L concentration of Ang II induced the expression of PlGF above basal levels, and the induction was peaked at 24 hours after treatment (Figure 1A). The induction of PlGF mRNA by Ang II was best at 10^-6 ^mol/L and was less effective at 10^-7 ^mol/L or 10^-5 ^mol/L (Figure 1B). Standard concentration of 10^-6 ^mol/L Ang II was used for all further experiments. Similarly, Ang II at 10^-6 ^mol/L also led to a time-dependent increase of PlGF mRNA expression in HUASMCs (Figure 1A).

Next, we examined the Ang II- induced change of PlGF protein level in EA.Hy 926 endothelial cells and HUASMCs. Cells were exposed to Ang II treatment for indicated times. Subsequently, the secretion of PlGF in the culture media was measured by ELISA. Under serum-starvation condition, EA.Hy 926 cells secreted low level of PlGF (~ 12 pg/mL). Treatment with Ang II for 8, 24 or 48 hours evoked significant secretion of PlGF, as compared to the untreated cells (Figure 1C). Similarly, the growth-arrested HUASMCs released low level of PlGF protein in the cell culture supernatant. When HUASMCs were treated with Ang II for 24 or 48 hrs, the PlGF production was significantly higher than that in untreated cells (Figure 1C).

The expression of PlGF was also analyzed by immunofluorence technique. Only weak PlGF fluorescence intensity was observed in growth-arrested EA.Hy 926 cells and HUASMCs. Ang II stimulation for 24 hours significantly increased the cytoplasmic PlGF fluorescence intensity in both cell types (Figure 1D).

Taken together, our data indicated that Ang II increased PlGF mRNA and protein production in EA.Hy 926 cells and HUASMCs.

### Angiotensin II increases VEGFR-1 and VEGFR-2 mRNA expression in both EA.Hy 926 cells and HUASMCs

**Figure 2:**
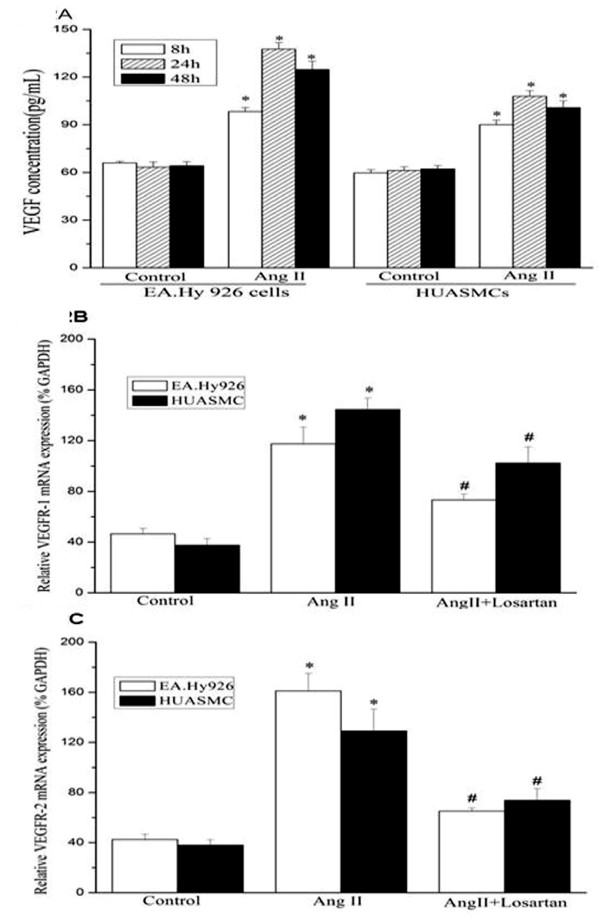
**Angiotensin II increases VEGF release (A) and VEGFR-1 and -2 mRNA expressions (B, C) in EA.Hy 926 endothelial cells and HUASMCs. (A)** Release of VEGF in the cell culture media measured by ELISA. Growth-arrested cells were incubated with Ang II (10^-7^, 10^-6 ^and 10^-5 ^mol/L) for 8 hrs, 24 hrsor 48 hrs, and VEGF protein released into cell culture media was measured by ELISA (n = 3 in each group). * indicates *P *< 0.05 *vs *control EA.Hy 926 cells or HUASMCs. **(B)** Real-time PCR results: Ang II induced VEGFR-1 mRNA expression in both EA.Hy 926 endothelial cells and HUASMCs. * indicates *P *< 0.05 *vs *control EA.Hy 926 cells or HUASMCs. # indicates *P *< 0.05 *vs *cells treated with Ang II. **(C)** Real-time PCR results: Ang II induced VEGFR-2 mRNA expression in both EA.Hy 926 endothelial cells and HUASMCs. * indicates *P *< 0.05 *vs *control EA.Hy 926 cells or HUASMCs. # indicates *P *< 0.05 *vs *cells treated with Ang II.

VEGF is a the major initiator of angiogenesis and its actions are mediated via two major receptors, VEGFR-1(Flt-1) and VEGFR-2(KDR) [24,25]. VEGF is an essential mediator in Ang II-induced vascular inflammation and structural changes through its proinflammatory actions [24]. In order to investigate the possible role of VEGF and its receptors in our experimental model, we first measured the release of VEGF in the culture supernatant of these cells. Our results showed that treatment with Ang II significantly increased the release of VEGF in the cell culture supernatant (Figure 2A). We also examined the VEGFR-1 and VEGFR-2 gene expression by real-time PCR. We showed that the expression of both VEGFR-1 and -2 mRNA were induced upon Ang II stimulation. This induction of VEGFR gene expression was abolished by the AT_1_R blocker, Losartan (Figure 2B and 2C). Our results confirmed that VEGF and its receptors might be involved in Ang II actions in VECs and VSMCs.

### Angiotensin II increases PlGF expression via AT_1_R in both EA.Hy 926 cells and HUASMCs

**Figure 3:**
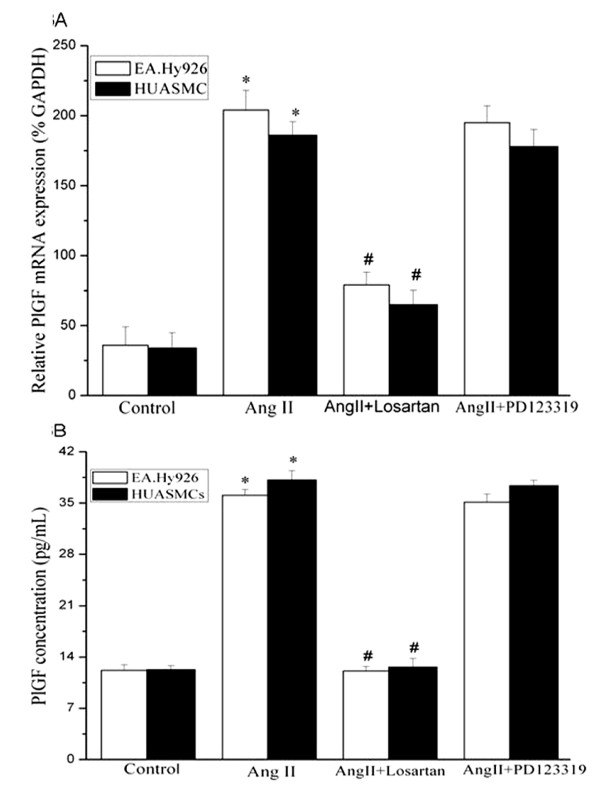
**Angiotensin II induces PlGF mRNA (A) and protein levels (B) via AT_1 _in EA.Hy 926 endothelial cells and HUASMCs**. Cells were pretreated for 30 min with 10^-6 ^mol/L Losartan (AT_1_R antagonist) or ^-6 ^mol/L PD123319 (AT_2_R antagonist) and then stimulated with 10^-6 ^mol/L Ang II for 24 hours. **(A) **Q-PCR results: PlGF mRNA expression was assayed by Q-PCR. Experiments were performed five times with the similar results (n = 5 in each group). * indicates *P *< 0.05 *vs *control EA.Hy 926 cells or HUASMCs. # indicates *P *< 0.05 *vs *cells treated with Ang II. **(B) **PlGF protein production in the cell culture media was measured by ELISA (n = 3 in each group). * indicates *P *< 0.05 *vs *control EA.Hy 926 cells or HUASMCs. # indicates *P *< 0.05 *vs *cells treated with Ang II.

In VECs and VSMCs, Ang II acts through two specific receptors, AT_1_R and AT_2_R [8]. To evaluate the roles of the two receptors in the Ang II-induced PlGF expression, EA.Hy926 cells and HUASMCs were pre-treated with the two receptor antagonists, and then incubated with Ang II. The AT_1_R antagonist Losartan caused a significant decrease in Ang II-induced PlGF expression at both mRNA and protein levels (Figure 3), whereas the AT_2_R antagonist PD 123319 had no effect, suggested that Ang II-inuced PlGF up-regulation was mediated through its AT_1 _receptor.

### Signaling mechanisms involved in Angiotensin II-induced PlGF gene and protein production

**Figure 4:**
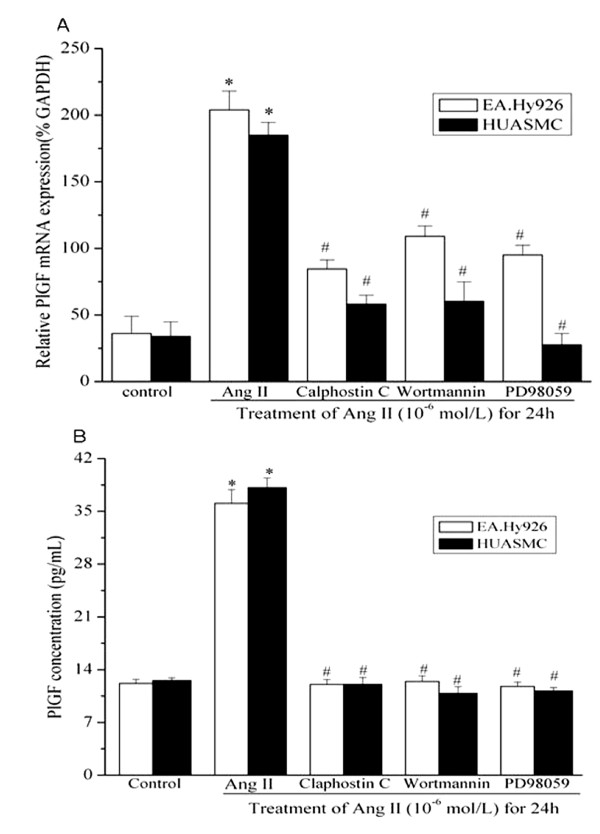
**Molecular mechanisms involved in Angiotensin II-induced PlGF upregulation in EA.Hy 926 cells and HUASMCs**. **(A)** Effects of different inhibitors on the PlGF mRNA expression induced by Ang II by using Quantitative RT-PCR. Cells were pretreated with different inhibitors for 30 mins and stimulated with Ang II (10^-6 ^mol/L) for 24 hours, and PlGF mRNA expression was assayed by Q-PCR (n = 3 in each group). * *P *< 0.05 *vs *control cells. # *P *< 0.05 *vs *cells treated with Ang II alone. **(B)** Effects of different inhibitors on the PlGF protein expression induced by Ang II detected by using ELISA. PlGF protein contents in the cell culture media were assayed by ELISA (n = 3 in each group). * *P *< 0.05 *vs *control cells. # *P *< 0.05 *vs *cells treated with Ang II alone.

Ang II, via AT_1_R, activates several intracellular signaling pathways, including PKC, ERK1/2 and PI-3K [26-28]. To evaluate the roles of downstream signaling in the Ang II-induced PlGF expression, EA.Hy926 cells and HUASMCs were pre-treated with kinase inhibitors Calphostin C, PD98059 or Wortmannin. By using quantitative RT-PCR and ELISA, subsequent signal inhibition experiments revealed that treatment with Calphostin C, PD98059, and Wortmannin significantly decreased the Ang II-induced PlGF mRNA and protein expression in the both cell types (Figure 4). These data suggest that, PKC, ERK1/2 and PI-3K all involved in stimulating PlGF expression by Ang II.

### Role of PlGF in the Angiotensin II-induced proliferation of EA.Hy 926 cells and HUASMCs

In order to examine if PlGF or VEGF played a role in Ang II-induced proliferation, we used MTT incorporation assay [29,30] to study the proliferation of growth-arrested EA.Hy 926 cells and HUASMCs followed by (1) Ang II treatment, or (2) co-treatment of Ang II and a neutralizing antibody against to PlGF or VEGF (10 or 20 μg/mL), or (3) administration of the blocking antibody for PlGF or VEGF alone for 24 h.

**Figure 5:**
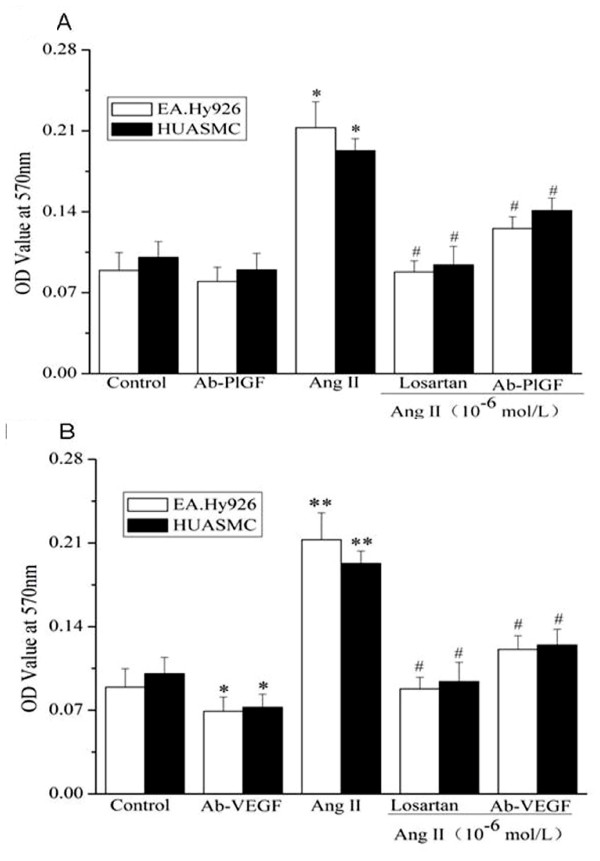
**PlGF and VEGF is involved in Angiotensin II-induced proliferation of cultured EA.Hy 926 cells and HUASMCs**. Quiescent cells were treated with Ang II (10^-6 ^mol/L), Ang II (10^-6 ^mol/L) and Losartan (10^-6 ^mol/L), Ang II (10^-6 ^mol/L) and the specific neutralizing antibody to PlGF**(A) **or VEGF **(B)**(20 μg/mL) for 24 hours, followed by the assessment of cell proliferation by MTT assay. Each value is the mean ± SD of 5 separate determinations. * indicates *P *< 0.05 *vs *control cells. # indicates *P *< 0.05 *vs *cells treated with Ang II. Ab-PlGF: the specific neutralizing antibody against PlGF. Ab-VEGF: the blocking antibody to VEGF.

Figure 5 shows that, similar to other reports[31,32] in which administration of Ang II stimulated the proliferation of cultured vascular cells, treatment with Ang II (10^-6 ^mol/L) for 24 hours significantly increased EA.Hy 926 cell proliferation by 138% compared to the control; and increased HUASMCs proliferation by 91.6%. A neutralizing antibody specific to PlGF inhibited Ang II-induced proliferation in EA.Hy 926 cells (40.9% inhibition), and to a less extent, in HUASMCs (26.8% inhibition) (Fig. 5A). Moreover, a neutralizing antibody specific to VEGF inhibited Ang II-induced proliferation in EA.Hy 926 cells (43.3% inhibition), and to a less extent, in HUASMCs (35.5% inhibition) (Fig 5B). Ang II-stimulated cell proliferation was suppressed by the simultaneous administration of the AT_1_R blocker Losartan (69.8% and 62.2% inhibition rate in EA.Hy 926 and HUASMCs respectively). However, administration of blocking antibody to PlGF alone had no effect on the proliferation in these vascular cells, while the blocking antibody to VEGF alone could significantly suppressed the proliferation rate in both EA.Hy 926 cells and in HUASMCs. Our data indicated that PlGF was involved in Ang II-induced proliferation of EA.Hy 926 cells and HUASMCs, whereas VEGF was implicated in both basal and Ang II-induced proliferation in these cells.

## Discussion and Conclusions

In the present study, the potential correlation between Ang II and PlGF was investigated in cultured EA.Hy 926 endothelial cells and in HUASMCs. The major findings of this study are: (1) Ang II, via AT_1_R, induces PlGF gene expression and protein secretion in both VECs and VSMCs; (2) Ang II increases VEGFR-1 and -2 gene expression in these vascular cells; (3) multiple signaling pathways, including PKC, ERK1/2 and PI3-K, are involved in this Ang II-induced PlGF upregulation; (4) blockade of PlGF results in the inhibition of Ang II-induced proliferation in these cells, whereas blockade of VEGF leads to inhibition of both basal and Ang II-elicited proliferation. Our observations established a role of PlGF in mediating Ang II-induced proliferation in vascular endothelial cells and vascular smooth muscle cells. In addition, our results suggested that inhibition of PlGF or VEGF might be useful in preventing abnormal VEC or VSMC proliferation evoked by Ang II.

Numerous studies suggest that the renin-angiotensin system (RAS) contributes to the pathogenesis of atherosclerosis [4,7]. Angiotensin II, the principal effecter of the RAS, is not only a vasoactive hormone, but also a cytokine that regulates cell proliferation, inflammation and fibrosis. Ang II elicits the inflammatory response by stimulating the production of chemokines, cytokines, and adhesion molecules [10]. Recent study demonstrated that Ang II induced vascular endothelial cell proliferation by increasing the expression of the angiogenic factor VEGF [31]. Consistent with previous reports [24,25,31], we observed that Ang II increased VEGF and its two receptors expression in our experimental model. Meanwhile, our data provided direct evidence that, in vascular endothelial cell and smooth muscle cells, Ang II, via its AT_1 _receptor, could up-regulate PlGF expression. These results suggested that Ang II might participate in the regulation of pathological angiogenesis.

Ang II acts through binding to its specific AT_1 _and AT_2 _receptors, which are seven transmembrane glycoproteins with 30% sequence similarity[8]. The AT_1_R is a classical G protein-coupled receptor, whereas AT_2_R often antagonizes the effects of signaling through the AT_1_R[9]. Many AT_1_R-induced growth responses are mediated by transactivation of growth factor receptors. AT_1 _receptor regulates cell proliferation, cytokines production and some pathological processes, including Ang II-induced hypertension and cardiac hypertrophy. Although AT_1 _receptor mediates most of the recognized cardiovascular effects of Ang II, the AT_2 _receptor contributes to the regulation of blood pressure and renal function. Our data demonstrated that, in cultured EA.Hy 926 endothelial cells and HUASMCs, Ang II increased PlGF expression and synthesis via its AT_1 _receptor.

Several pathways, e.g. PKC [26], ERK1/2 [7,26,33], and PI3K/Akt [34], involved in AT_1_R activation. Using specific inhibitors, the present study showed that Ang II activated PKC, ERK1/2 and PI-3K pathways. The activation of all these pathways contributed to PlGF up-regulation in EA.Hy 926 endothelial cells and HUASMCs.

The induction of PlGF gene expression by Ang II may be of considerable clinical significance, especially in vascular inflammation and atherosclerosis. Pro-inflammatory cytokines play a crucial role of in the development of atherosclerosis and plaque instability. Quiescent vascular endothelial cells only release minimal amounts of PlGF. In contrast, activated endothelial cells could produce large amounts of PlGF[35]. Previous studies demonstrated that PlGF activated monocytes and increased the expression of tumor necrosis factor-α(TNF-α), interleukin-1β (IL-1β), and monocyte chemotactic protein-1(MCP-1) in monocytes[36,37]. Consequently, when stimulated by Ang II, vascular endothelial cells produce PlGF, which activates neutrophils and monocytes, results in their adherence to endothelial cells. This might trigger the pathophysiological changes observed in atherosclerosis. Our results indicated that, the administration of PlGF-neutralizing antibody significantly inhibited the Ang II-dependent proliferation of vascular endothelial cells, suggested that PlGF might be a down-stream angiogenic mediator of RAS. The neutralizing antibodies of PlGF or VEGF are less effective in inhibiting cell proliferation than the small molecule inhibitor of AT_1_R, since other effects are involved besides VEGF/PlGF production. It has been largely demonstrated that the Ang II-induced VSMC proliferation is mediated by PDGF and Egr-1 [38,39]. And the AT_1_R blocker might be useful as an anti-angiogenic agent.

It has been recognized that VSMC proliferation within the vessel wall is an important pathogenic feature in the development of atherosclerosis [40,41]. Ang II has been implicated to play an important role in this cellular mechanism. When exposed to hypoxia (3% O_2_), the proliferation and contraction of VSMC were enhanced by PlGF treatment [42]. Furthermore, recent study has shown that PlGF expression in human atherosclerotic carotid plaques is related to inflammation and clinical plaque instability[12]. Present observations showed that Ang II induced PlGF expression in VSMC, suggested a role of PlGF in mediating VSMC proliferation induced by Ang II.

It has been shown that Ang II could stimulate the expression of hypoxia inducible factor-1α (HIF-1α) [43], and HIF-1α seems to be involved in the enhanced PlGF expression stimulated by hypoxia [19]. The hypoxia-inducible PlGF expression is mediated through NF-κB, metal-regulatory transcription factor-1(MTF-1) and the interaction between them [19]. Ang II-induced PlGF expression might be mediated through the HIF-1α pathways. However, future studies in vascular cells are necessary to determine the role of Ang II receptors and PlGF in atherosclerosis.

In conclusion, PlGF might be one of the downstream effectors up-regulated by Ang II in vascular diseases. Several pathways, such as PKC and ERK 1/2 activation, seem to be involved in the Ang II-induced PlGF expression in vascular cells. PlGF also mediates Ang II-induced cell proliferation in VECs and HUASMCs. Thus, our study provides new insights into PlGF as one of the Ang II-inducible genes. The link of PlGF to Ang II might be a novel molecular mechanism to target cardiovascular diseases.

## Methods

### Cell Culture

A human endothelial cell line (EA.Hy 926) was grown in RPMI 1640 (GIBCO-BRL) supplemented with 10% FBS, 1% penicillin/streptomycin, 2 mmol/L L-glutamine and 1% HAT (Sigma, USA).

Primary cultures of HUASMCs were isolated from freshly delivered umbilical cords by tissue explanting method [22,23] and maintained in DMEM medium (GIBCO-BRL) supplemented with 20% fetal bovine serum (FBS) (Hyclone), 2 mmol/L L-glutamine, and 1% penicillin-streptomycin. All cell cultures were maintained in a humidified 5% CO_2_/95% air incubator at 37°C. HUASMCs were identified by the specific marker of vascular smooth muscle cell (α-smooth muscle actin, α-SMA) immunofluorescence. The study was approved by the medical ethics committee of the West China Hospital, Sichuan University.

In all experiments, confluent EA.Hy 926 cells or HUASMC cells at passage 4 to 8 were washed and incubated with serum-free media for 24 hours. These cells were treated with human angiotensin II (Ang II, 10^-6 ^mol/L) for different time points. In some experiments, cells were pre-treated for 30 minutes with 10^-7 ^mol/L Wortmannin, 10^-6 ^mol/L Calphostin C, 10^-6 ^mol/L PD98059, 10^-6 ^mol/L Losartan (AT_1_R antagonist) or 10^-6 ^mol/L PD123319(AT_2_R antagonist), and then stimulated with 10^-6 ^mol/L Ang II for different time points.

### Quantitative reverse transcription PCR (Real-time RT-PCR)

The expression of PlGF gene was identified by quantitative RT-PCR (Q-PCR) as reported earlier [44]. Total RNA was extracted from HUASMCs or EA.Hy 926 cells using TRIZOL reagent (Invitrogen, USA). Q-PCR was carried out on an ABI Prism 7300 PCR Detection System (Applied Biosystems, USA) with fluorescence dye SYBR Green (SYBR Green Real-time PCR Master Mix, TOYOBO, Japan). The sequences of the primers were as follows: PlGF-F: 5'-GTT CAG CCC ATC CTG TGT CT-3'; PlGF-R: 5'-CTT CAT CTT CTC CCG CAG AG-3'. GAPDH-F: 5'-ACC ACA GT CCA TGC CAT CAC-3'; GAPDH-R: 5'-TCC ACC ACC CTG TTG CTG TA-3'. VEGFR-1-F: 5'-ATC ATT CCG AAG CAA GGT GTG-3'; VEGFR-1-R: 5'-AAA CCC ATT TGG CAC ATC TGT-3'. VEGFR-2-F: 5'-CAC CAC TCA AAC GCT GAC ATG TA-3'; VEGFR-2-R: 5'-GCT CGT TGG CGC ACT CTT-3'. The thermal cycling conditions were as following: 95°C 60 seconds, 40 cycles of 95°C for 15 seconds, 56°C for 15 seconds, 72°C for 45 seconds (data collection). Data analysis was carried out by ABI sequence detection software using relative quantification. For quantification, the target sequence was normalized to the GAPDH mRNA levels.

### Quantification of PlGF and VEGF by ELISA

The PlGF or VEGF levels in the cell culture supernatant were measured by a "sandwich" enzyme immunoassay (Quantikine ELISA, R&D Systems, USA) according to manufacture's instructions. Briefly, the samples were added to 96-well plates coated with a specific monoclonal antibody to PlGF or VEGF. The unbound protein was washed three times, and an enzyme-linked polyclonal antibody specific to PlGF or VEGF was added. The plates were washed again for three times, and substrate solution was added to the wells. After 30 min of incubation, stop solution was added to each well. The amount of PlGF or VEGF was determined by optical density of the samples by comparison with the standards at 450 nm using an ELISA reader (Bio-Rad Model 680, USA).

### Detection PlGF protein expression by immunofluorescence

EA.Hy 926 cells or HUASMCs were plated onto coverslips in 6-well plates, growth arrested and treated with Ang II at 10^-6 ^mol/L concentration with or without other compounds. After that, the cells on the glass slides were then fixed and blocked as described before [31], followed by exposed to the primary anti-PlGF antibody. The second antibody was FITC-conjugated antibody and the cell nuclei were stained with DAPI (Sigma-Aldrich). Images were collected using an Eclipse TE2000-U fluorescent microscope system (Nikon, Japan) and analyzed with ImageJ software from NIH Image to semi-quantitatively determine the expression of PlGF protein in the cytoplasm of the cells.

### Assessment of cell proliferation

The effect of Ang II and antagonists to PlGF or VEGF on cell proliferation was determined by the 3-(4,5-dimethylthiazol-2-yl)-2,5-diphenyltetrazolium bromide (MTT) assay, as described previously[29]. Briefly, EA.Hy 926 cells or HUASMC cells were subcultured in 96-well plates and incubated with serum-free medium for 24 hours. Quiescent cultures were treated with the Ang II (10^-6 ^mol/L), Ang II (10^-6 ^mol/L) and Losartan (10^-6 ^mol/L), Ang II (10^-6 ^mol/L) and the specific PlGF or VEGF neutralizing antibody (10-20 μg/mL) for 24 hours. Twenty microliters of MTT (15 mg/mL, Sigma, USA) was added to each well and incubated for 4 h at 37°C. The culture supernatant was discarded by aspiration and 150 μL of dimethyl sulfoxide (DMSO, Sigma) was added for 10 min. The light absorbance at 570 nm was detected using ELISA reader (Bio-Rad Model 680, USA).

### Statistical analysis

The experimental data were expressed as means ± SD. Group means were compared by *One-way ANOVA *using the statistical software program SPSS 10.0 for Windows (Chicago, IL, USA), and *P *value < 0.05 was considered significant in all cases.

## Authors' contributions

PP and HF conceived of the experiments, carried out all experiments and prepared the manuscript. LZ and HH conceived of the experiments and performed real-time RT-PCR. FL conceived of the experiments. WW performed cell culture. YG and XL provided expert advice and interpretation of the study's results. All authors read and approved the final manuscript.
